# Gene delivery to breast cancer by incorporated EpCAM targeted DARPins into AAV2

**DOI:** 10.1186/s12885-023-11705-5

**Published:** 2023-12-11

**Authors:** Ya-feng Lv, Hao Zhang, Zhi Cui, Cui-jiao Ma, Yu-ling Li, Hua Lu, Hong-yan Wu, Jian-lin Yang, Chun-yu Cao, Wen-zheng Sun, Xiao-fei Huang

**Affiliations:** 1 Hubei Key Laboratory of Tumor Microenvironment and Immunotherapy, College of Basic Medical Sciences, China Three Gorges University, No. 8 Da Xue Road, Yichang City, Hubei Province China; 2 Department of Pathology, Qilu Hospital, Shandong University, Shandong, China

**Keywords:** Adeno-associated viruses, Breast Cancer, EpCAM, DARPins

## Abstract

**Objective:**

The aim of this study is to evaluate an AAV vector that can selectively target breast cancer cells and to investigate its specificity and anti-tumor effects on breast cancer cells both in vitro and in vivo, offering a new therapeutic approach for the treatment of EpCAM-positive breast cancer.

**Methods:**

In this study, a modified AAV2 viral vector was used, in which EpCAM-specific DARPin EC1 was fused to the VP2 protein of AAV2, creating a viral vector that can target breast cancer cells. The targeting ability and anti-tumor effects of this viral vector were evaluated through in vitro and in vivo experiments.

**Results:**

The experimental results showed that the AAV2M^EC1^ virus could specifically infect EpCAM-positive breast cancer cells and accurately deliver the suicide gene HSV-TK to tumor tissue in mice, significantly inhibiting tumor growth. Compared to the traditional AAV2 viral vector, the AAV2M^EC1^ virus exhibited reduced accumulation in liver tissue and had no impact on tumor growth.

**Conclusion:**

This study demonstrates that AAV2M^EC1^ is a gene delivery vector capable of targeting breast cancer cells and achieving selective targeting in mice. The findings offer a potential gene delivery system and strategies for gene therapy targeting EpCAM-positive breast cancer and other tumor types.

**Supplementary Information:**

The online version contains supplementary material available at 10.1186/s12885-023-11705-5.

## Introduction

In recent years, there has been a significant advancement in the use of adeno-associated virus (AAV)-based gene drugs for treating human monogenic diseases, resulting in unprecedented therapeutic effects [1–3]. This breakthrough has also paved the way for utilizing AAV vectors in anti-tumor gene therapy [4–6]. One major challenge that remains is the efficient and targeted delivery of gene drugs to tumor cells. AAV serotypes have natural tissue tropisms, such as AAV2 and AAV8 being liver-tropic, and AAV5 and AAV6 being lung-tropic [7–9]. However, when AAV vectors are systemically administered, they tend to accumulate in the liver, limiting their ability to effectively transduce other target tissues [10]. Moreover, local administration can lead to vector leakage into the bloodstream, causing systemic spread of the AAV capsid antigen and transgene [11]. Thus, the development of tumor-targeted AAV vectors has emerged as a prominent area of focus in the field of gene therapy.

The epithelial cell adhesion molecule (EpCAM) is highly expressed in various tumor cells, including those of breast cancer [12]. Prior research has indicated that EpCAM-specific DARPins, a unique type of target-binding protein, have the ability to bind to EpCAM with a high degree of affinity [13]. Utilizing this knowledge, a novel tumor-targeted AAV vector called EpCAM-AAV was developed by incorporating these EpCAM-specific DARPins directly into the AAV2 capsid [14]. This EpCAM-AAV was proven to effectively identify EpCAM-positive breast cancer cells, specifically MDA-MB-453, in an in vitro setting. In a similar vein, Alexander Muik and his team developed a technique to covalently attach DARPins to the surface of adenovirus-associated viral (AAV) particles through the use of protein trans-splicing(PTS) [15]. This PTS-coupled AAV also successfully recognized EpCAM-positive breast cancer cells in vitro. However, questions remain as to whether the DARPins-modified AAV2 vector can specifically identify tumor cells in an in vivo environment, and whether the application of this vector for therapeutic gene delivery can effectively impede the growth of tumor cells.

In this study, we adopted a similar methodology, integrating the EpCAM-specific DARPin EC1 into the AAV2 capsid, which led to the creation of an AAV vector named AAV2M^EC1^. Our research demonstrated the ability of AAV2M^EC1^ to selectively infect breast cancer cells in mice and effectively transport the suicide gene HSV-TK, leading to the suppression of tumor growth. These findings emphasize the promising prospects of AAV2M^EC1^ as a cutting-edge vector for delivering genes in breast cancer treatment. Additionally, our results open up new avenues for the development of targeted gene delivery systems and innovative strategies for anti-tumor gene therapy.

## Methods

### The construction of AAV vector

In order to eliminate the affinity of the heparin sulfate receptor for AAV2, we obtained the pRC2M plasmid by point mutation (R585A, R588A) at position 585 and 588 of VP1 in pRC2 plasmid [16]. Using the pRC2M plasmid as a template, we generated the plasmid pRVP1/3 M (providing VP1 and VP3 with the R585A and R588A mutations.) by point mutation of the VP1 protein at the T138A position through PCR. Similarly, using the pRC2M plasmid as a template, we mutated the VP1 protein at positions M1L and M203L to obtain the plasmid pVP2M. Subsequently, we obtained the plasmid pEC1-VP2M by cloning the synthesized EC1 sequence [13](synthesized by Vazyme, Suzhou, China) into the N-terminal of the VP2 within the pRC2M plasmid by homologous recombination (clone smarter technologies, USA) (sense: 5’-GAGGAACCTGTTAAGATGGACCTGGGCAAGAAG-3’ and antisense: 5’-GGATCCGCCGCCGCCGGATCCGCCGCCGCCGCTGCCGCCGCCGCCGTTCAGCT-3’). The His-tag was inserted into the N-terminal of EC1 in the pEC1-VP2M plasmid, named pHis-EC1-VP2M (sense:5’-CTTCTTGCCCAGGTCCATCTTAACAGGTTCCTC-3’and antisense: 5’-GGCGGCGGCGGATCCGGCGGCGGCGGATCCGCTCCGGGAAAAAAGAGGCCGGTA-3’). In addition, two AAV expression plasmids (pAAV-LUC and pAAV-HSV-TK) were produced. The method was as follows: PCR was used to amplify the luciferase gene, which was then inserted into the pAAV-GFP plasmid by enzymatic ligation (replacing the GFP coding sequence), the enzymatic cleavage sites were *Eco*RI and *Hind*III (Thermo Fisher Scientific Inc). The same approach was taken to produce the pAAV-HSV-TK plasmid.

### Preparation and purification of AAV particles

AAV was produced as previously described [17]. Briefly, for production of the AAV2M^EC1^ vectors, HEK-293T cells were transfected with plasmids pRVP1/3 M,

pEC1-VP2M, pHelper, and the vector plasmid in a ratio of 6:6:10:5. For production of the AAV2 vectors, HEK-293T cells were transfected with plasmids pRC2, pHelper, and the vector plasmid in a ratio of 8:10:6. For production of the AAV2M vectors, HEK-293T cells were transfected with plasmids pRVP1/3 M, pVP2M, pHelper, and the vector plasmid in a ratio of 6:6:10:5. For production of the AAV2M^His − EC1^ vectors, HEK-293T cells were transfected with plasmids pRVP1/3 M, pHis-EC1-VP2M, pHelper, and the vector plasmid in a ratio of 6:6:10:5. The medium supernatant and cell sediment were harvested after 72 h, followed by three cycles of freeze-thaw lysis. The resulting lysate was then centrifuged at 2,000 rpm for 10 min, and the supernatant was mixed with the culture medium supernatant before adding proportional amounts of PEG8000. After centrifugation at 4,000 rpm for 60 min, the virus precipitate was resuspended with PBS, treated with benzonase to digest remaining DNA and RNA, and obtained by low-speed centrifugation. The crude virus solution was further purified by density gradient centrifugation using iodixanol density gradient solution to obtain AAV virus as described previously [17].

### QPCR detection for AAV titers

All the packaged AAV were taken and treated at 95℃ for 10 min, then used as template. ITR sequence of the virus (sense: 5’- CTCAATGGGTGGAGTATTT-3’and antisense: 5’- GAGTGAAGCAGAACGTGGG − 3’) as amplification primers. The assay was performed on the real-time quantitative PCR instrument (Analytik Jena AG, Germany) according to the following procedure: initial denaturation 95 °C for 3 min, 39 cycles of amplification were performed under following condition: 95 °C for 10 s, 60 °C for 30 s. The virus titer was calculated from the standard curve obtained by multiplying the Ct value and copy number of the diluted standard, as previously described [17].

### Western blot identification of viral expression

2 × 10^10 AAV genomic particles were separated by electrophoresis on a 10% SDS-PAGE gel and transferred onto a polyvinylidene fluoride (PVDF) membrane. To enhance antibody hybridization efficiency, the membrane was appropriately cropped in areas without samples before the hybridization process. Primary antibodies against viral capsid proteins (vp1, vp2, and vp3) or His-tag were used for detection at a dilution of 1:1000. The primary antibodies used were anti-capsid protein antibody (03-61058, ARP) or anti-His-tag antibody (M20001, Abmart). The membrane was then incubated with HRP-conjugated goat anti-mouse secondary antibodies (1:3000, Servicebio, Wuhan, Hubei). The immune-reactive bands were detected using a chemiluminescent method (Pierce ECL assay, Thermo Fisher). Due to the cropping of the membrane, the full-length membrane cannot be displayed.

### ELISA assay for EC1 protein displayed on the surface of the virus

To investigate the surface distribution of the EC1 protein in AAV2M^EC1^ viruses, we employed a double antibody sandwich method to detect AAV2M^EC1 − his^ and AAV2 viruses. In brief, polystyrene plates were coated with His-tag antibody, and various dilutions of AAV2 and AAV2M^EC1 − his^ viruses were added to the plates. The captured AAV viruses were then bound to anti-Capsid antibody. Visualization of the results was achieved by adding chromogenic solution and substrate, followed by measuring the absorbance at 450 nm using an Automated ELISA System (JM-850, shjingmi, China).

### In vitro analysis

To compare the infection efficiency of AAV2, AAV2M, and AAV2M^EC1^ viruses on 4T-1 cells in vitro, 4T-1 was placed in a 12-well plate and incubated overnight at 37 °C and 5% CO_2_. AAV (MOI = 1 × 10^5^) was added and infected for 48 h. Images were collected by inverted fluorescence microscopy.

### In vivo analysis

All mice were purchased from the Experimental Animal Center of Three Gorges University. To investigate the targeting of the experimental virus and the anti-tumor activity of the expressed gene HSV-TK (preserved in our laboratory), 4T-1 cells (2.5 × 10^5^) were implanted subcutaneously into the buttocks of BALB/c female mice [18]. When tumor volume of the mice reached 3 mm^3^, the animals were randomly divided into three groups and separately treated by I.V. injection with 5 × 10^11^vg of AAV2, AAV2M and AAV2M^EC1^ virus. After 2 days, ganciclovir (GCV) was given intraperitoneally at an injectable dose of 100 mg/kg once every 2 days [6]. The tumor volume size and weight of each animal was measured every 2 days during the experiment. The tumor volumes were calculated as follows: tumor volume = (length × width^2^)/2. The mice were sacrificed 15 days post-AAV treatment. The tumors were photographed and weighed.

In vivo bioluminescence imaging: Detection of in vivo targeting of AAV2M^EC1^ on day 7 of AAV injection in tumor-bearing mice. 20 mg/kg dose of D-luciferin (Yeasen, shanghai, china) was injected intraperitoneally into the mice. Then luminescence signal was collected in the animals by the IVIS (*Lumina XRMS*, America) within 10–20 min. In addition, the organs harvested from the mice are imaged. All the data were analyzed using the imaging software.

Processing of samples: Liver and tumor samples were harvested after decortication of all surviving mice. The samples were fixed overnight in 4% paraformaldehyde solution, then dehydrated, embedded and prepared into paraffin and sectioned to a thickness of 4 μm for subsequent experiments.

Immunohistochemistry: These sections were then subjected to autoclave thermal repair in citric acid sodium citrate buffer, followed by inactivation of endogenous peroxidase with 3% H_2_O_2_. 10% goat serum is used to enclose tissues for 30 min at 37 °C, then the sections were incubated overnight at 4 °C with primary antibody luciferase antibody (1:500, ab185924, abcam). HRP-conjugated goat anti-mouse (1:200, servicebio, Wuhan, Hubei) was incubated for 1 h at room temperature as a secondary antibody. The samples were detected by DAB (3,3′-Diaminobenzidine tetrahydrochloride). Microscopic observation of stained sections and quantitative analysis using software.

Hematoxylin and eosin: Liver sections were stained with hematoxylin and eosin using an automatic staining machine. Finally, the slices were sealed with neutral resin for histopathological evaluation.

### Statistical analysis

Statistics and graphs were prepared with Prism 8 software. The normality of data distribution was tested with the Shapiro–Wilk normality test. For more than two groups, we performeded a one-way analysis of variance (ANOVA), followed by independent two-tailed t-tests between the two groups to compare differences in the data. All immunoblotting was performed with technical replicates of at least 3. A p value of < 0.05 was considered statistically significant.

## Results

### The production and identification of AAV2M^EC1^ virus

**Fig. 1:**
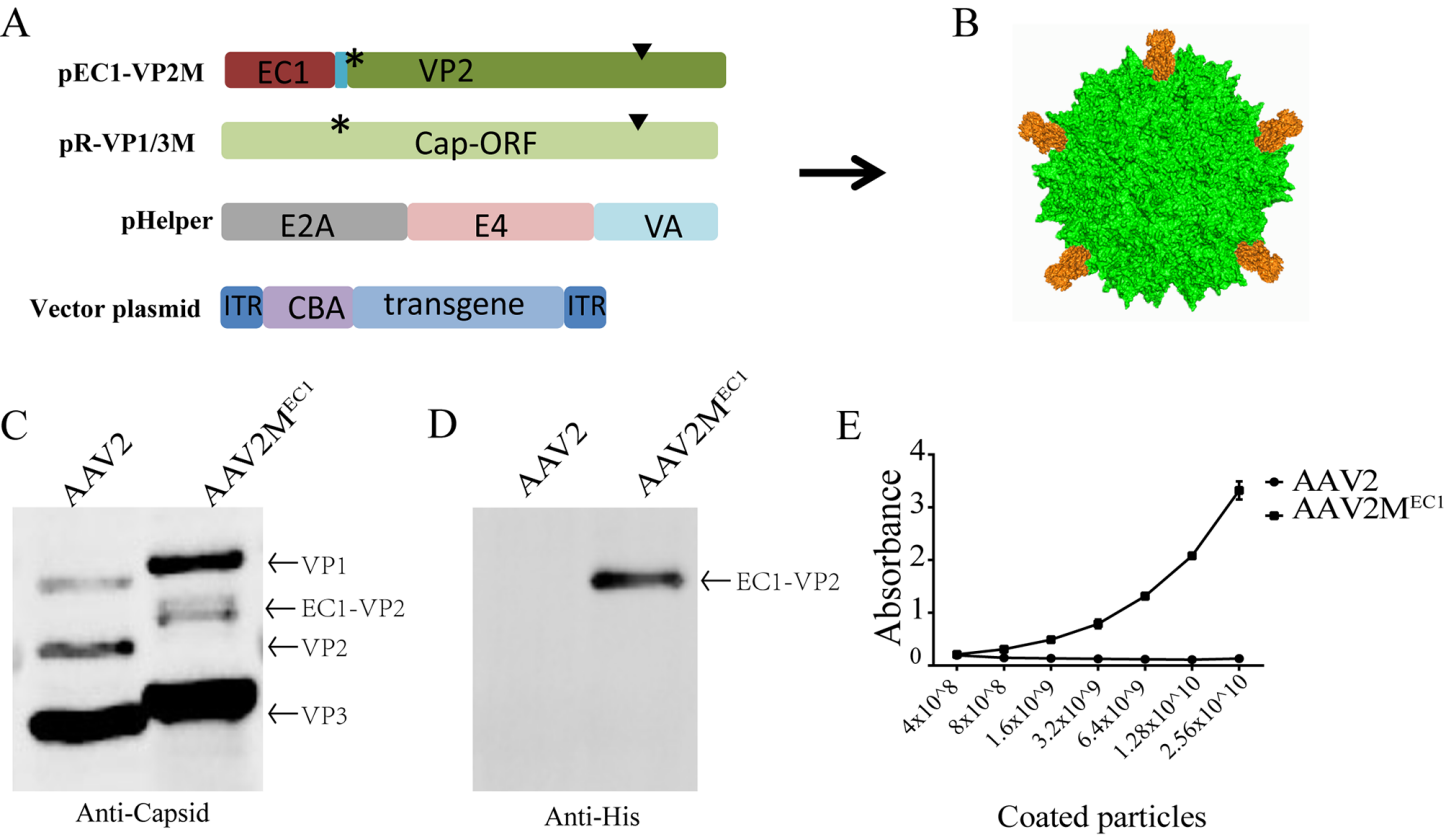
The production and identification of AAV2M^EC1^ virus. **A**: In plasmids pEC1-VP2M and pR-VP1/3 M, the start codon of VP2 was mutated (labeled by asterisk) to prevent its expression. To eliminate binding to HSPG, R585 and R588 in pEC1-VP2M and pR-VP1/3 M were replaced with alanine (labeled by triangle). **B**: Schematic illustration of the AAV2M^EC1^ capsid are shown. Five DARPin molecules (yellow) extending from the five-fold symmetry axis of the AAV2M^EC1^ capsid, Illustration was created using Pymol and PDB files 6IH9 and 5KNG. **C**: Western blot analysis of AAV2M^EC1^ virus. The fusion of DARPin molecule EC1 with VP2 protein led to a reduced mobility of AAV capsid proteins in SDS-PAGE. **D**: His-tag was inserted at the N-terminus of the EC1 sequence and packaged into virus, which was detected using His-tag antibody to detect EC1 protein in the virus. **E**: ELISA was used to determine EC1 display on the virus surface. AAV2M^His − EC1^ virus was able to bind to plates coated with His-tag antibody, while AAV2 virus was used as a control and the bound virus particles were quantified using Cap-specific antibody. N = 3 experiments; mean ± SD.

In this research, to eliminate the tropism of AAV2 to the liver and other non-target tissues, we replaced arginine residues with alanine at positions R585 and R588 of VP1, which are the two primary residues responsible for HSPG binding [16, 18]. As reported, insertion of GFP or DARPin protein coding sequence at the N-terminus of VP2 did not affect virus packaging efficiency, but conferred the AAV virus with new infection capabilities [18, 19]. Therefore, the N-terminus of AAV capsid protein VP2 is a suitable insertion site for proteins. To redirect tropism, a new plasmid named pEC1/VP2M was constructed by inserting DARPin EC1, which binds EpCAM with high affinity [13], at the N-terminus of VP2 (Fig. 1A). The schematic illustration of the AAV2M^EC1^ is shown in Fig. 1B. The plasmids used for virus packaging are listed in Supplementary Table 1, AAV2M^EC1^ viruses with titers comparable to the control AAV2 virus, as determined by qPCR (Supplementary Table 1). We then used Western blot to detect AAV capsid protein, which showed that VP1, VP2, and VP3 could be detected in wild-type AAV2 and targeted AAV2M^EC1^ virus. Due to the fusion expression of EC1 and VP2 protein, the speed of protein migration was affected, resulting in the up-shift of EC1-VP2 bands in electrophoresis and the relative abundance of VP1, VP2 and VP3 in the AAV2M^EC1^ viral particles are altered compared with the parental AAV2 virus (Fig. 1C). This result is consistent with previous reports [18, 19]. The reason for this change is currently unclear, but it is possible that the insertion of EC1 has altered the composition of AAV viral capsid proteins, thereby affecting the overall composition of the AAV virus.

The key to tumor targeting is that the targeted EC1 protein must be displayed on the surface of the virus. First, we fused a His tag to the N-terminus of the EC1 protein, and Western blot analysis confirmed the presence of the His tag in the AAV2M^EC1^ virus (Fig. 1D). Next, we added different dilutions of AAV2 and AAV2M^EC1^ viruses to a polystyrene reaction plate coated with His antibody, and finally added AAV Capsid antibodies (Fig. 1E). Only AAV2M^EC1^ virus was captured by the His antibody and generated a signal, and its signal strength increased with increasing virus titer. AAV2 viruses only produced a basal signal. Therefore, the above experiments demonstrate that we successfully inserted the EC1 sequence into VP2 of AAV2, and the His tag can be displayed on the surface of AAV2.

### The AAV2M^EC1^ exhibits targeting specificity towards Tumor cells

**Fig. 2:**
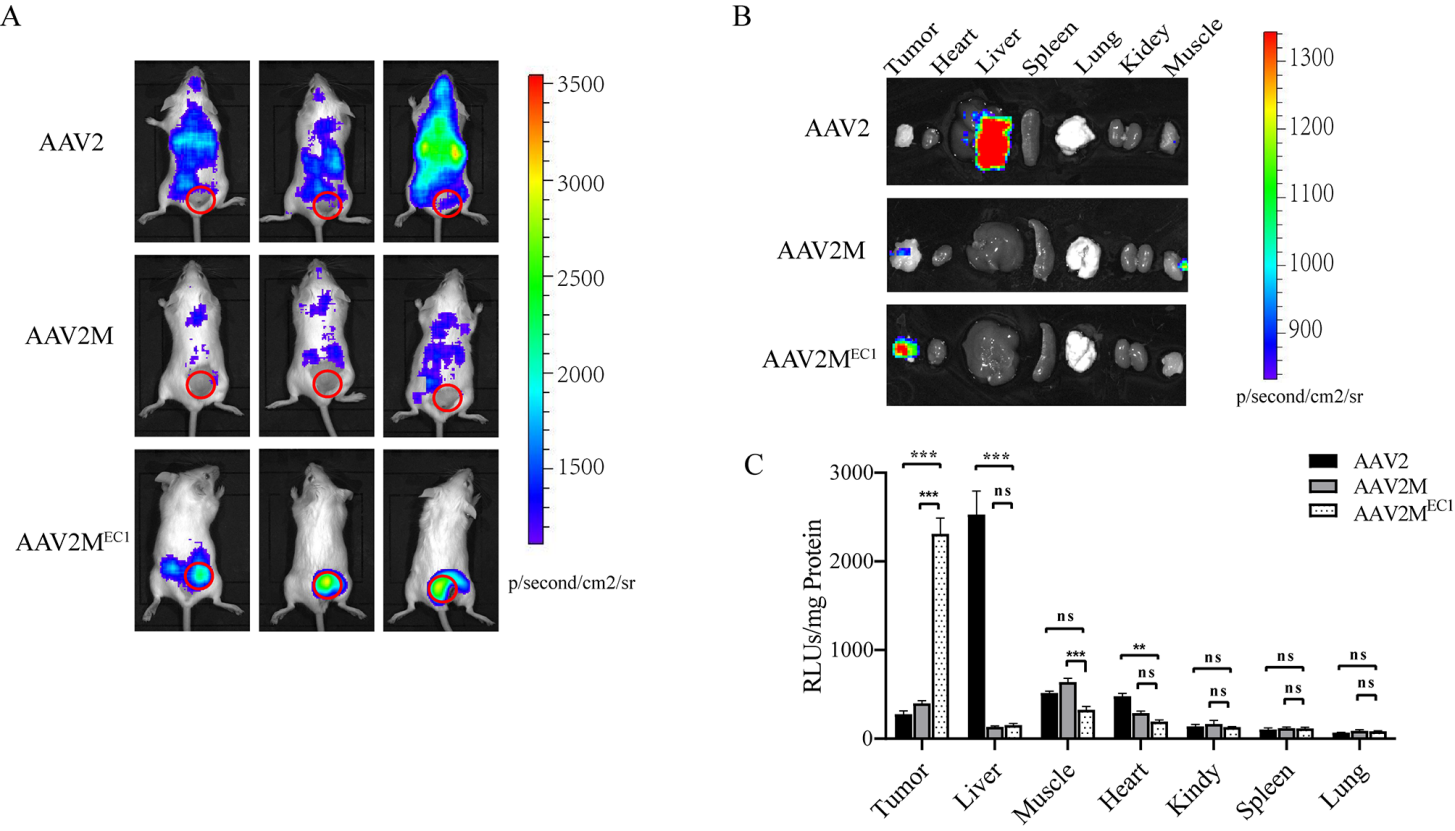
AAV2M^EC1^ virus targets EpCAM-positive tumors in vivo. **A**: Bioluminescence imaging of 4T-1 tumor in mice. The mice were divided into three groups and were respectively injected with AAV2, AAV2M, and AAV2M^EC1^ vector, with a single dosage of 1 × 10^11 AAV particles per mouse via intravenous injection. On day 7 after injection, the bioluminescence imaging was conducted to detect the expression of luciferin in the mice using an in vivo imaging system. The red circles show the sites of tumor cell injections. **B**: Ex vivo bioimaging of organs in mice from each group (tumor, heart, liver, spleen, lung, kidney, and muscle). The luminescence signal intensity was expressed as photons/second/square centimeter/steradian (p/sec/cm2/sr). **C**: Luciferase activity assay indicates targeted delivery of the gene to the tumor. Two weeks after injection, the mice were euthanized, and multiple tissues (liver, heart, lung, kidney, spleen, skeletal muscle, and tumor) were collected for quantitative luciferin measurement. The luciferase activity are reported as relative light units per mg tissue (RLUs/mg tissue). Data are presented as mean ± SD. *P < 0.05; **P < 0.01; ***P < 0.001; ns statistically not significant

**Fig. 3:**
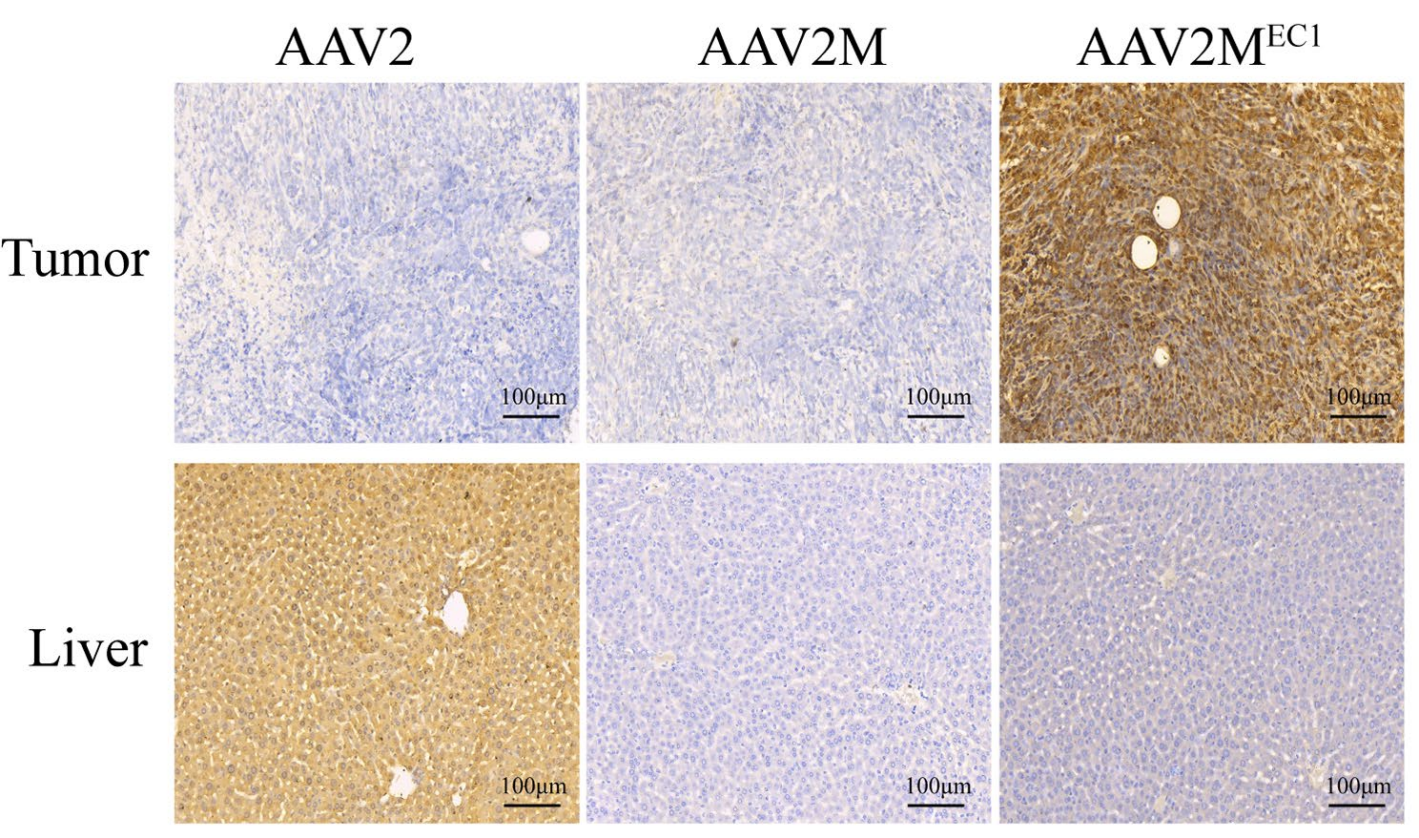
Distribution of AAV2M^EC1^ in 4T1 tumor xenografts and liver of mice. Immunohistochemistry for luciferase was performed on tumor and liver sections to characterize the distribution of targeted gene delivery. Two weeks after AAV administration, paraffin sections of tumor and liver were prepared and processed for luciferase detection by immunohistochemistry. Luciferase-expressing cells were stained golden-brown in color. Scale bar represents 100 μm

To evaluate the targeted transduction capability of AAV2M^EC1^ in vivo on EpCAM-positive breast cancer cells 4T-1, we subcutaneously injected 2.5 × 10^5 4T-1 cells into the back of each BALB/c female mice. A single tail vein injection was performed with AAV2, AAV2M and AAV2M^EC1^ virus carrying a luciferase gene at a dose of 1 × 10^11vg. After 7 days, bioluminescent imaging analysis showed that mice injected with AAV2 had a wide distribution of signals, primarily concentrated in the abdomen. The luminescence signals in the AAV2M group were distributed throughout the body, but with weak intensity. In contrast, all luminescence signals in the AAV2M^EC1^ group were concentrated at the tumor site with strong signal intensity, and no signals were observed in other areas (Fig. 2A). Imaging of peeled mouse tissues and organs showed that the luminescence signals in the AAV2 group mainly originated from the liver, with signal intensity 9 times that of the AAV2M^EC1^ group. For the signals in the tumor, the AAV2M^EC1^ group was 3 times that of the AAV2 group. On the other hand, due to a mutation in the HSPG binding site of AAV2M, AAV2M no longer has a natural affinity for liver tissue. Therefore, this group of mice only had weak signals in the tumor and muscle tissues (Fig. 2B and Supplementary Fig. 1). Quantitative analysis of luciferase activity in organ lysates confirmed the in vivo imaging data, showing that the AAV2M^EC1^ group had the highest luciferase activity in the tumor, which was 10 times higher than the AAV2 group and AAV2M group (Fig. 2C). Immunohistochemical analysis of EpCAM expression in tumor and liver tissue slices showed that EpCAM expression in tumor tissue was positive(Supplementary Fig. 2), which was consistent with the positive expression of luciferase detected only in the tumor slices of the AAV2M^EC1^ group (Fig. 3). This indicates that the AAV2M^EC1^ virus has the ability to target and infect EPCAM-positive breast cancer cells.

To evaluate the in vitro targeting infectivity of AAV2M^EC1^, we changed from luciferase to GFP-expressing vectors and infected 4T-1 cells for 48 h. The results showed that the infectivity of AAV2M^EC1^ in 4T1 cells is lower than that of AAV2 but higher than AAV2M, indicating that the insertion of the DARPin can partially rescue the infectivity of AAV2M^EC1^ in 4T1 cells (Supplementary Fig. 3). In summary, these results demonstrate that the AAV2M^EC1^ vector exhibits targeted transduction capability in vivo and in vitro for EpCAM-positive breast cancer cells.

### In vivo antitumor effects without liver toxicity

**Fig. 4:**
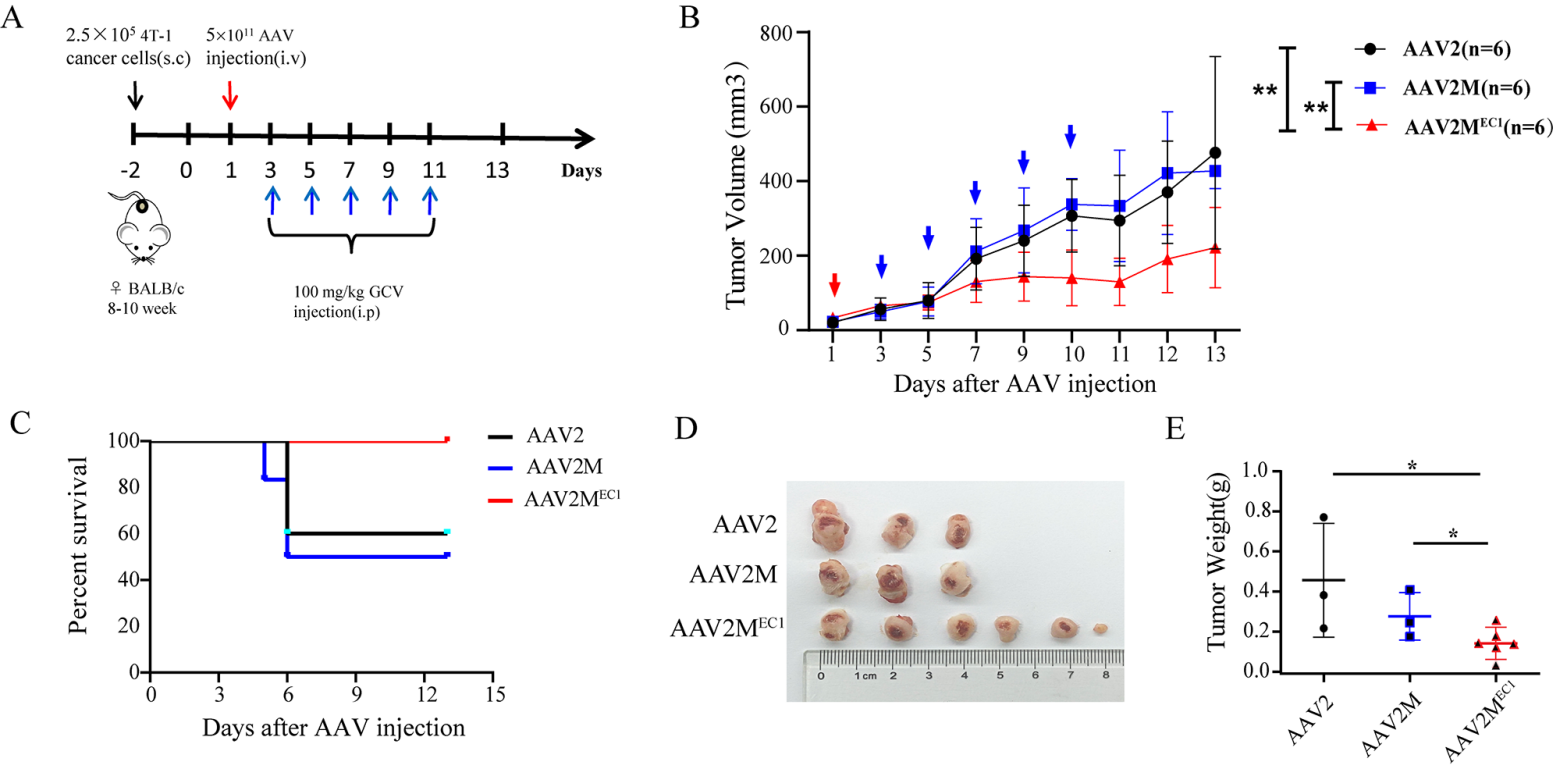
AAV2M^EC1^-mediated HSV/TK gene transfer reduces the growth of 4T1 tumor xenografts. **A**: Schematic showing experimental design in vivo. Female BALB/c mice were subcutaneously injected with 2 × 10^5 4T1 cells in the buttocks. On the third day after tumor inoculation, the mice were divided into three groups. A single intravenous injection of 5 × 10^11 viruses was administered, followed by intraperitoneal administration of GCV (100 mg/kg) 2 days later. This was repeated once every other day for a total of 5 times. The groups included AAV2 (n = 6), AAV2M (n = 6) and AAV2M^EC1^ (n = 6). **B**: Measured tumor volumes. The data show that the AAV2M^EC1^ tumors grew more slowly than the AAV2 and AAV2M tumors. The red arrow indicates the time point of virus injection, and the blue arrow indicates the time point of GCV injection. Tumor volume (mm^3^) is represented as mean ± SD. **C**: Percentage survival of AAV2 treated mice (black), AAV2M (blue), AAV2M^EC1^ (red). **D**: Representative images of tumors after each mouse was euthanized。**E**: Accumulated tumor weights are shown for mice receiving AAV2, AAV2M and AAV2MEC1-treated. Data are presented as mean ± SD. *P < 0.05; **P < 0.01; ns statistically not significant

We engineered AAV2MEC1 to carry the herpes simplex virus thymidine kinase (HSV-TK), a suicide gene capable of converting the prodrug ganciclovir (GCV) into cytotoxic compounds that induce cell killing [20, 21]. Mice with subcutaneous 4T-1 tumors were intravenously injected with equal amounts of AAV2, AAV2M, and AAV2M^EC1^ particles carrying the HSV/TK gene (Fig. 4A). The tumor volumes in the AAV2 and AAV2M groups grew at nearly identical rates, while the tumor growth rate in the AAV2M^EC1^ group significantly slowed from day 5 onwards (Fig. 4B). There were no differences in body weight among all groups of mice (Supplementary Fig. 3). During the GCV treatment period, some mice in the AAV2 and AAV2M groups died, resulting in survival rates of 60% and 50% respectively, whereas all mice in the AAV2M^EC1^ group survived throughout the observation period (Fig. 4C). The cause of mortality in the AAV2M group of mice may be attributed to off-target effects or unintended systemic responses to the AAV2M treatment. Although liver transduction was limited in this group, it is possible that other organs or systems were affected by the AAV2M treatment, leading to the observed mortality. The tumor weight of mice in the AAV2M^EC1^ group was significantly lower than that of mice in the AAV2 and AAV2M groups (Fig. 4D and E).

**Fig. 5:**
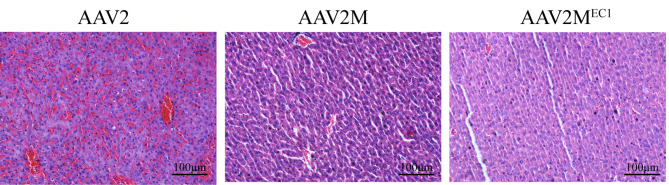
H&E staining of liver slices for the different groups. A representative image is shown. Dilation of central vein and surrounding sinusoids, filled with a large number of red blood cells, was observed in the liver lobules of mice from AAV2 group but not in AAV2M and AAV2M^EC1^ groups. Scale bar represents 100 μm

We performed hematoxylin and eosin analysis on liver tissue from the three groups of mice. The results revealed significant dilation of the central vein and surrounding sinusoids in the liver lobules of mice in the AAV2 group, accompanied by a large number of red blood cells (Fig. 5). This observation may be attributed to cell damage caused by the suicide gene delivered by AAV2, which primarily targeted the liver. This finding may explain the lower survival rate observed in the AAV2 group. No evident liver toxicity was observed in the AAV2M and AAV2MEC1 groups of mice.

## Discussion

The concept of using AAV vectors for gene therapy has been extensively studied and has shown promising results in the treatment of various diseases [22]. AAV vectors have been utilized to deliver therapeutic genes, including suicide genes, immune checkpoint blockers, microRNA, and tumor suppressor genes, to inhibit tumor cell proliferation and migration [4–6]. However, the efficient and targeted delivery of therapeutic genes to tumor cells remains a challenge. AAV serotypes have natural tissue tropisms, such as liver-tropism for AAV2, which limits their transduction efficiency in other target tissues. To overcome this limitation, researchers have explored different strategies to develop tumor-targeted AAV vectors.

One approach is to modify the AAV capsid to redirect its tropism towards tumor cells. The epithelial cell adhesion molecule (EpCAM) is highly expressed in various tumor cells, including breast cancer cells. Previous studies have shown that EpCAM-specific DARPins, a type of target-binding protein, can bind to EpCAM with high affinity. By fusing EpCAM-specific DARPins to the AAV2 capsid, researchers have successfully developed tumor-targeted AAV vectors, such as EpCAM-AAV [14] and PTS-coupled AAV [15]. These vectors have demonstrated the ability to recognize EpCAM-positive tumor cells in vitro. In addition, the anti-EpCAM-AAV2 vector is generated by linking anti-EpCAM antibody to AAV using a biotin-avidin bridge [23]. Both in vitro and in vivo experiments have demonstrated the vector’s ability to specifically target EpCAM-positive tumor cells. However, the process of viral preparation becomes more complex due to the need for conjugation between AAV and avidin, followed by the connection with biotinylated antibodies. Moreover, the anti-EpCAM-AAV2 vector, which contains biotin, avidin, and antibodies, has a larger molecular weight, increasing the potential risk of immune responses.

In our study, we took a similar approach by incorporating the EpCAM-specific DARPin EC1 into the AAV2 capsid. We successfully generated the AAV2M^EC1^ vector, which specifically infected EpCAM-positive breast cancer cells in mice (Figs. 2 and 3). Our in vivo experiments showed that AAV2M^EC1^ effectively delivered the suicide gene HSV-TK to tumor tissue, resulting in the inhibition of tumor growth (Fig. 4). Importantly, AAV2M^EC1^ exhibited reduced accumulation in liver tissue compared to the traditional AAV2 vector, minimizing potential liver toxicity.

While our study builds upon previous research on tumor-targeted AAV vectors, it offers several unique contributions. First, we demonstrated the selective targeting ability of AAV2M^EC1^ in vivo, providing evidence of its potential clinical application. Second, we successfully delivered the therapeutic gene HSV-TK to tumor tissue, leading to the inhibition of tumor growth. These findings highlight the potential of AAV2M^EC1^ as a vector for breast cancer gene delivery and provide new possibilities for the development of tumor-targeted gene delivery vehicles and anti-tumor gene therapy strategies.

In conclusion, our study introduces AAV2M^EC1^, a gene delivery vector specifically designed to target breast cancer cells. We have demonstrated its targeting ability and anti-tumor effects in vitro and in vivo. Building upon existing literature, our study provides new insights and advancements in breast cancer gene therapy. Further research is warranted to optimize the vector design and explore its potential clinical applications.

### Electronic supplementary material

Below is the link to the electronic supplementary material.

Supplementary Material 1

Supplementary Material 2

## Data Availability

The data underlying this article are available in the article and in its online supplemental material. All other supporting data are available from the corresponding authors on reasonable request.

## Abbreviations

AAVs: Adeno-associated viruses
EpCAM: Epithelial cell adhesion molecule
DARPin: Designed ankyrin repeat proteins
HSV-TK: Herpes simplex virus- thymidine kinase
VP1: Virus protein 1
VP2: Virus protein 2
VP3: Virus protein 3
qPCR: Quantitative polymerase chain reaction
GFP: Green fluorescent protein
GCV: Ganciclovir
HSPG: Heparan sulfate proteoglycans
CD3: Cluster of differentiation 3
ITR: Inverted terminal repeat
ELISA: Enzyme-linked immunosorbent assay
MOI: Multiplicity of infection
DAB: 3,3′-Diaminobenzidine tetrahydrochloride

## References

[1] Li C, Samulski RJ (2020). Engineering adeno-associated virus vectors for gene therapy. Nat Rev Genet.

[2] Tai CH, Lee NC, Chien YH, Byrne BJ, Muramatsu SI, Tseng SH, Hwu WL (2022). Long-term efficacy and safety of eladocagene exuparvovec in patients with AADC deficiency. Mol Ther.

[3] Pupo A, Fernández A, Low SH, François A, Suárez-Amarán L, Samulski RJ (2022). AAV vectors: the Rubik’s cube of human gene therapy. Mol Ther.

[4] Komoll RM, Hu Q, Olarewaju O, von Döhlen L, Yuan Q, Xie Y, Tsay HC, Daon J, Qin R, Manns MP (2021). MicroRNA-342-3p is a potent tumour suppressor in hepatocellular carcinoma. J Hepatol.

[5] GuhaSarkar D, Neiswender J, Su Q, Gao G, Sena-Esteves M (2017). Intracranial AAV-IFN-β gene therapy eliminates invasive xenograft glioblastoma and improves survival in orthotopic syngeneic murine model. Mol Oncol.

[6] Pan JG, Zhou X, Zeng GW, Han RF (2012). Potent antitumour activity of the combination of HSV-TK and endostatin armed oncolytic adeno-associated virus for Bladder cancer in vitro and in vivo. J Surg Oncol.

[7] Sands MS (2011). AAV-mediated liver-directed gene therapy. Methods Mol Biol.

[8] Kurosaki F, Uchibori R, Sehara Y, Saga Y, Urabe M, Mizukami H, Hagiwara K, Kume A (2018). AAV6-Mediated IL-10 expression in the lung ameliorates Bleomycin-Induced Pulmonary Fibrosis in mice. Hum Gene Ther.

[9] Liang SQ, Walkey CJ, Martinez AE, Su Q, Dickinson ME, Wang D, Lagor WR, Heaney JD, Gao G, Xue W (2022). AAV5 delivery of CRISPR-Cas9 supports effective genome editing in mouse lung airway. Mol Ther.

[10] Nathwani AC, Rosales C, McIntosh J, Rastegarlari G, Nathwani D, Raj D, Nawathe S, Waddington SN, Bronson R, Jackson S (2011). Long-term safety and efficacy following systemic administration of a self-complementary AAV vector encoding human FIX pseudotyped with serotype 5 and 8 capsid proteins. Mol Ther.

[11] Maturana CJ, Chan A, Verpeut JL, Engel EA (2023). Local and systemic administration of AAV vectors with alphaherpesvirus latency-associated promoter 2 drives potent transgene expression in mouse liver, kidney, and skeletal muscle. J Virol Methods.

[12] Gires O, Pan M, Schinke H, Canis M, Baeuerle PA. Expression and function of epithelial cell adhesion molecule EpCAM: where are we after 40 years? Cancer Metastasis reviews. 2020; 39(3):969–87. 10.1007/s10555-020-09898-3 PMC7497325 32507912

[13] Stefan N, Martin-Killias P, Wyss-Stoeckle S, Honegger A, Zangemeister-Wittke U, Plückthun A (2011). DARPins recognizing the tumor-associated antigen EpCAM selected by phage and ribosome display and engineered for multivalency. J Mol Biol.

[14] Münch RC, Muth A, Muik A, Friedel T, Schmatz J, Dreier B, Trkola A, Plückthun A, Büning H, Buchholz CJ (2015). Off-target-free gene delivery by affinity-purified receptor-targeted viral vectors. Nat Commun.

[15] Muik A, Reul J, Friedel T, Muth A, Hartmann KP, Schneider IC, Münch RC, Buchholz CJ (2017). Covalent coupling of high-affinity ligands to the surface of viral vector particles by protein trans-splicing mediates cell type-specific gene transfer. Biomaterials.

[16] Opie SR, Warrington KH, Agbandje-McKenna M, Zolotukhin S, Muzyczka N (2003). Identification of amino acid residues in the capsid proteins of adeno-associated virus type 2 that contribute to heparan sulfate proteoglycan binding. J Virol.

[17] Tian G, Cao C, Li S, Wang W, Zhang Y, Lv Y (2023). rAAV2-Mediated restoration of GALC in neural stem cells from Krabbe patient-derived iPSCs. Pharmaceuticals (Basel).

[18] Münch RC, Janicki H, Völker I, Rasbach A, Hallek M, Büning H, Buchholz CJ (2013). Displaying high-affinity ligands on adeno-associated viral vectors enables Tumor cell-specific and safe gene transfer. Mol Ther.

[19] Warrington KH, Gorbatyuk OS, Harrison JK, Opie SR, Zolotukhin S, Muzyczka N (2004). Adeno-associated virus type 2 VP2 capsid protein is nonessential and can tolerate large peptide insertions at its N terminus. J Virol.

[20] Angelici B, Shen L, Schreiber J, Abraham A, Benenson Y (2021). An AAV gene therapy computes over multiple cellular inputs to enable precise targeting of multifocal hepatocellular carcinoma in mice. Sci Transl Med.

[21] Pulkkanen KJ, Parkkinen JJ, Laukkanen JM, Kettunen MI, Tyynela K, Kauppinen RA, Ala-Opas MY, Yla-Herttuala S (2001). HSV-tk gene therapy for human renal cell carcinoma in nude mice. Cancer Gene Ther.

[22] Mendell JR, Al-Zaidy SA, Rodino-Klapac LR, Goodspeed K, Gray SJ, Kay CN, Boye SL, Boye SE, George LA, Salabarria S (2021). Current clinical applications of in vivo gene therapy with AAVs. Mol Ther.

[23] Lee S, Ahn HJ (2019). Anti-EpCAM-conjugated adeno-associated virus serotype 2 for systemic delivery of EGFR shRNA: its retargeting and antitumor effects on OVCAR3 Ovarian cancer in vivo. Acta Biomater.

