# Supplementary material for: Gene delivery to breast cancer by incorporated EpCAM targeted DARPins into AAV2
Source: BMC Cancer. 2023 Dec 11;23:1220. doi: 10.1186/s12885-023-11705-5 (PMC10712102; doi:10.1186/s12885-023-11705-5)

Figure 1C. Anti-Capsid

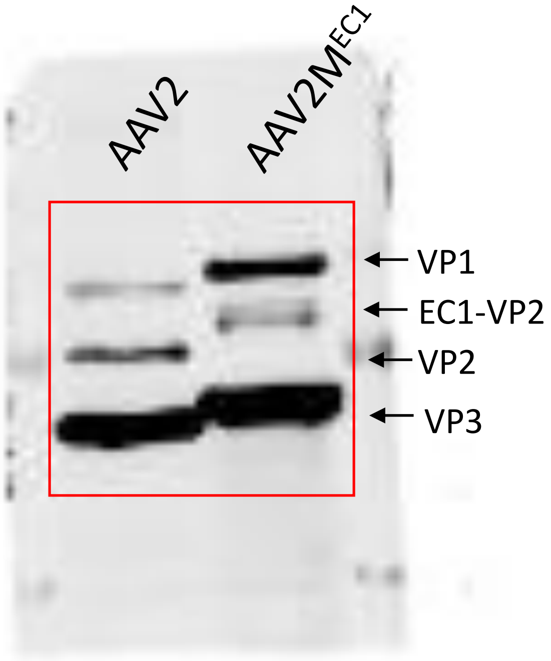

Anti-Capsid(replicates)

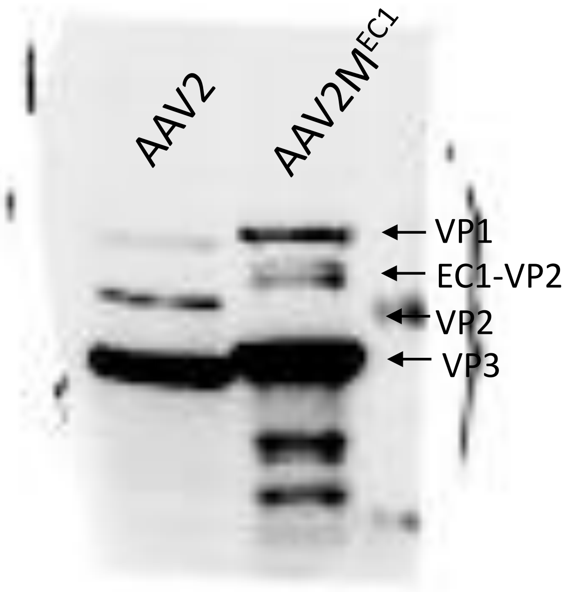

Anti-Capsid(replicates)

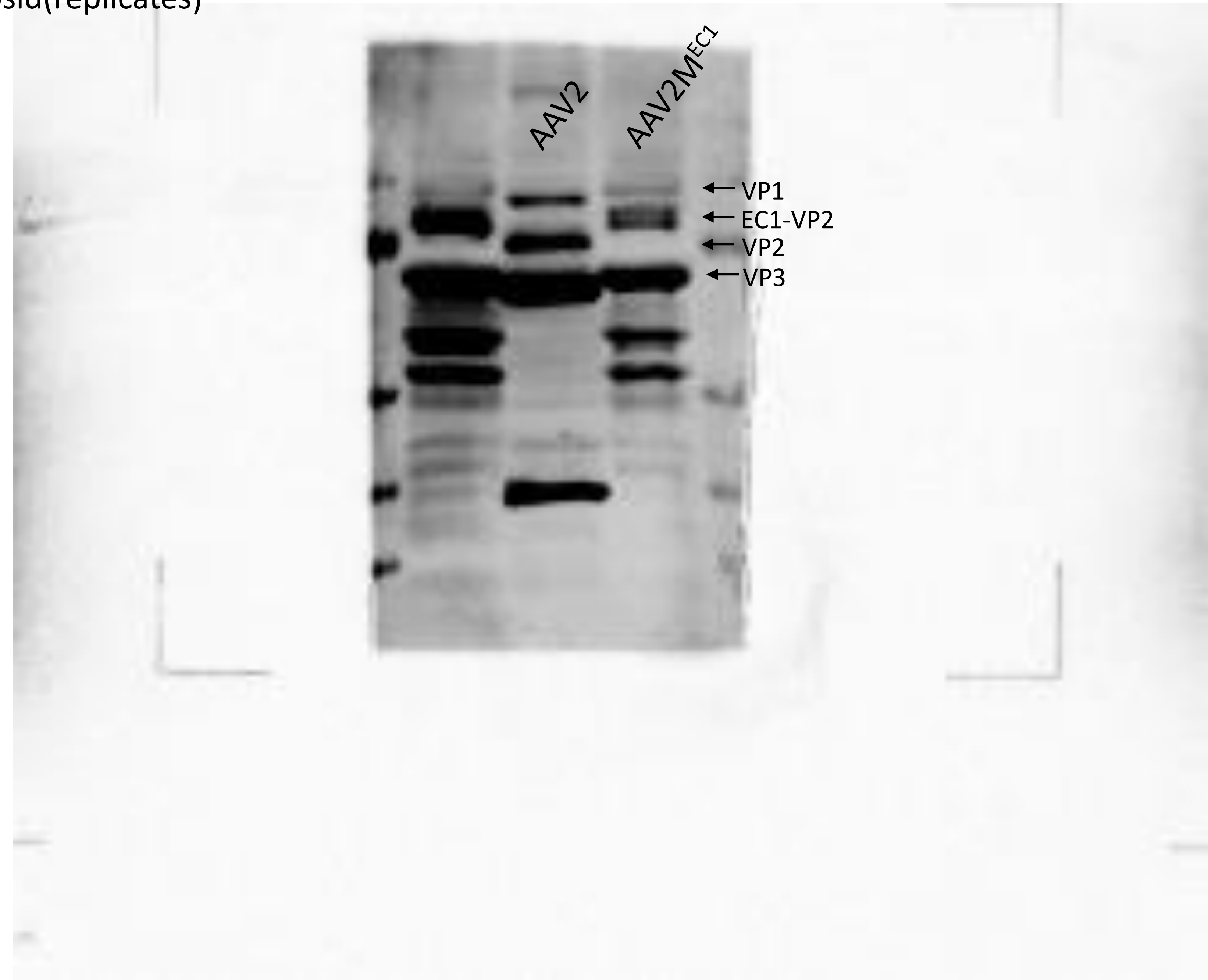

Figure 1D. Anti-His

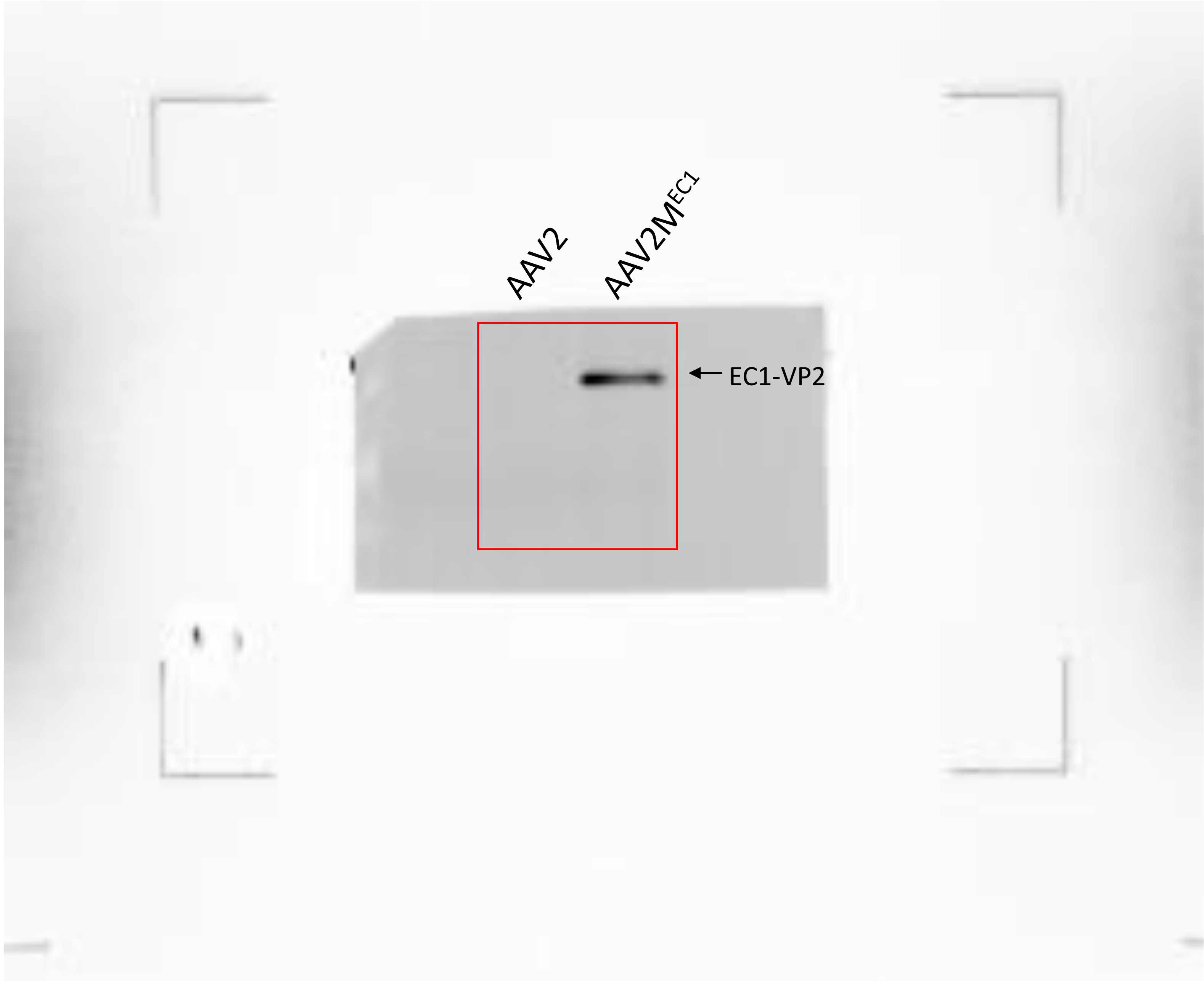

Anti-His(replicates)

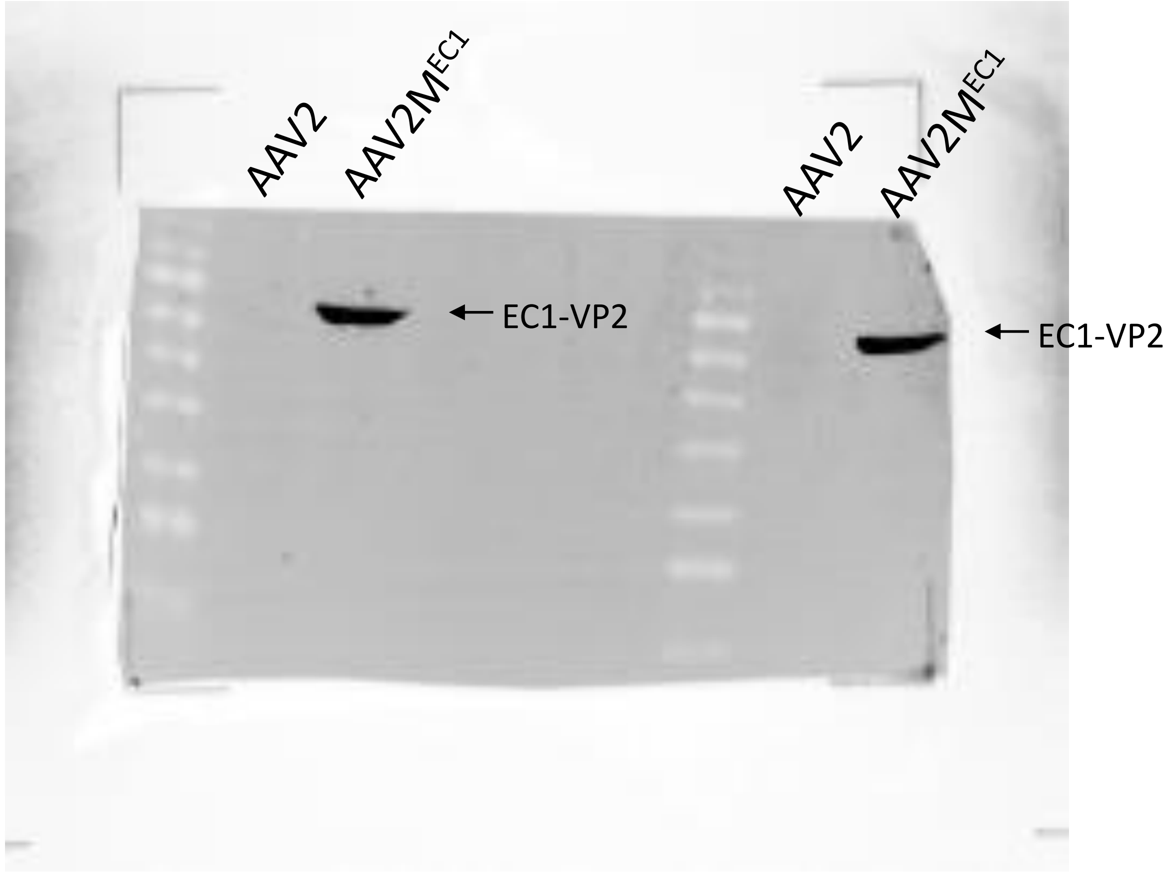

Supplement: Supplementary file 1 — Supplementary Material 1 [file 12885_2023_11705_MOESM1_ESM.pdf]
