# Supplementary material for: Gene delivery to breast cancer by incorporated EpCAM targeted DARPins into AAV2
Source: BMC Cancer. 2023 Dec 11;23:1220. doi: 10.1186/s12885-023-11705-5 (PMC10712102; doi:10.1186/s12885-023-11705-5)

Supplementary table 1

The plasmids used for AAV packaging and AAV titers.

**AAV serotypes**	**plasmids**	**Genomes/μl**
AAV2-GFP	pRC2, pHelper, pAAV-GFP	3.2×10^9
AAV2M-GFP	pRC2M, pHelper, pAAV-GFP	3.4×10^9
AAV2M^{EC1}GFP	pRVP1/3M, pEC1/VP2M, pHelper, pAAV-GFP	2.9×10^9
AAV2-LUC	pRC2, pHelper, pAAV-LUC	1.4×10^9
AAV2M-LUC	pRC2M, pHelper, pAAV-LUC	1.8×10^9
AAV2M^{EC1}LUC	pRVP1/3M, pEC1/VP2M, pHelper, pAAV-LUC	1.6×10^9
AAV2-TK	pRC2, pHelper, pAAV-TK	4.2×10^9
AAV2M-TK	pRC2M, pHelper, pAAV-TK	4.5×10^9
AAV2M^{EC1}TK	pRVP1/3M, pEC1/VP2M, pHelper, pAAV-TK	5.1×10^9

Supplementary figure 1

Quantification of the luminescence signals of indicated organs. mice were sacrificed immediately after *in vivo* imaging, the organs harvested from the executed mice are imaged by the IVIS. Quantification of the luminescence signals of tumor, liver, and muscle tissues. The AAV2M^{EC1} group was 3 times higher than the AAV2 and AAV2M group in tumors. On the other hand, due to the mutation of HSPG binding site in the AAV2M, the AAV2M and AAV2M^{EC1} groups only had weak signals in liver tissues. Data are presented as mean ± SD. **P < 0.01; ns statistically not significant.

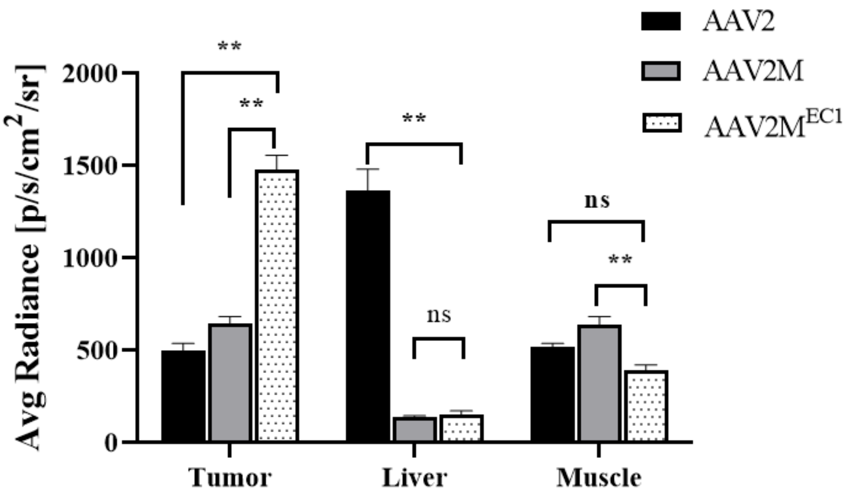

Supplementary figure 2

Immunohistochemical analysis of EpCAM expression in tumor and liver tissue slices showed that EpCAM expression in tumor tissue was positive. Scale bar represents 100 μm.

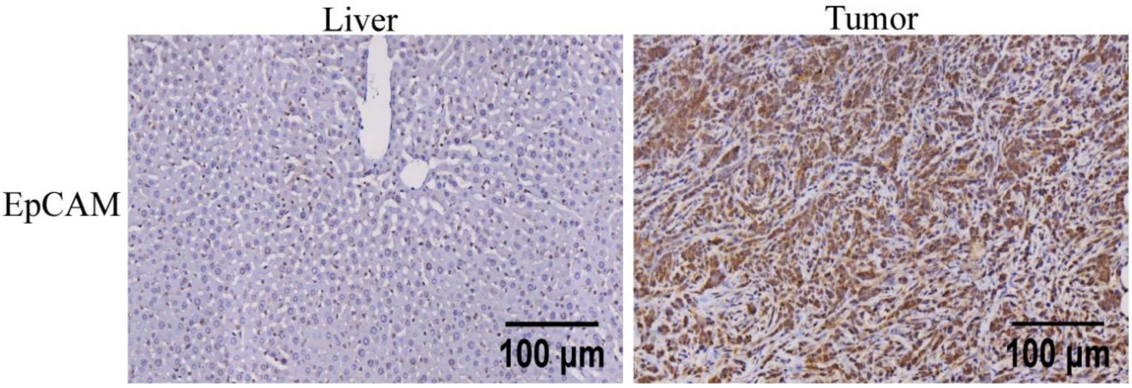

Supplementary figure 3

The infectivity of AAV2M^{EC1} virus for 4T-1 cells *in vitro*. The results showed that the infectivity of AAV2M^{EC1} in 4T1 cells is lower than that of AAV2 but higher than AAV2M. Scale bar represents 100 μm.

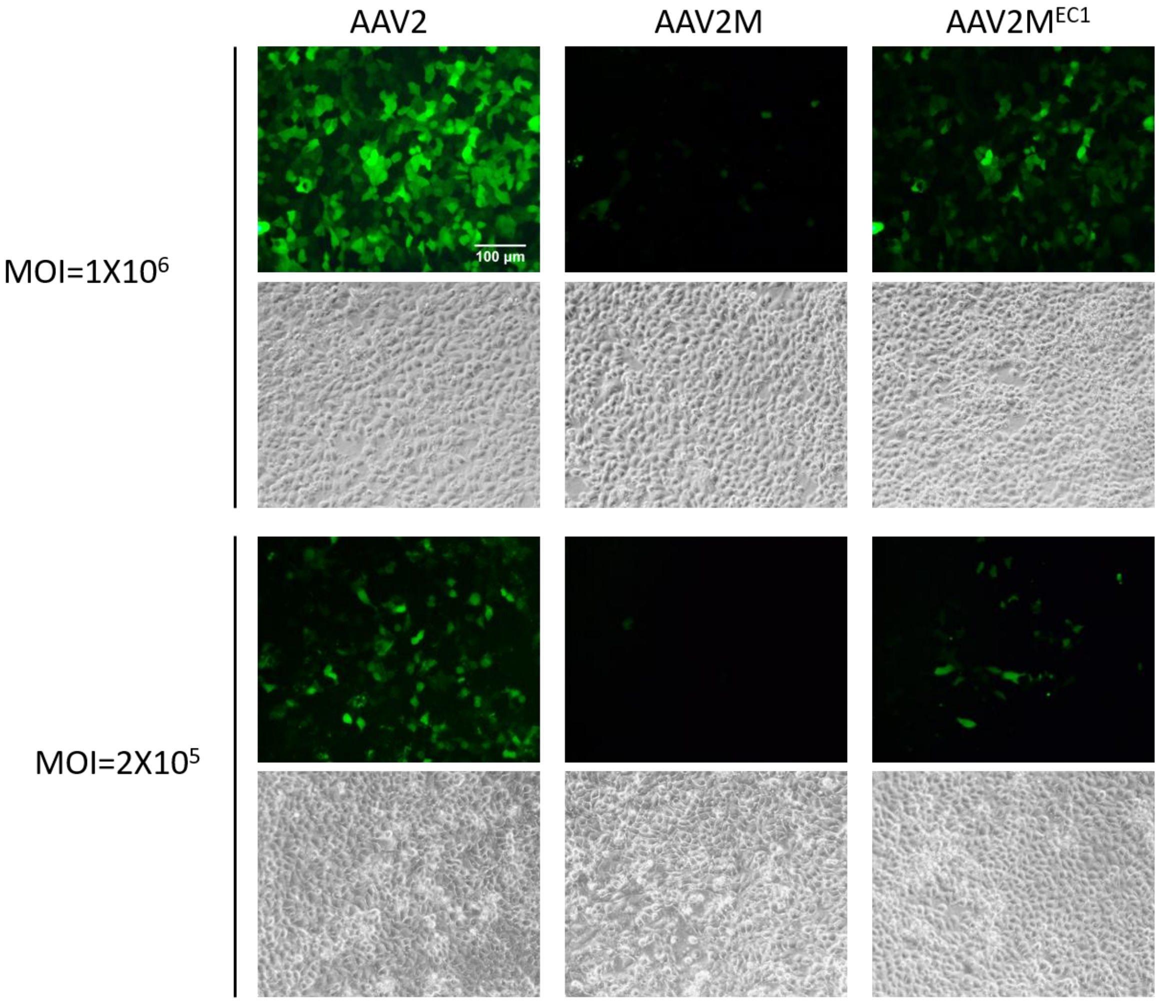

Supplementary Figure 4

Body weight of mice after AAV injection. There was no significant difference in body weight among the AAV2 (n=6), AAV2M (n=6) and AAV2M^{EC1} (n=6) group of mice. ns statistically not significant.

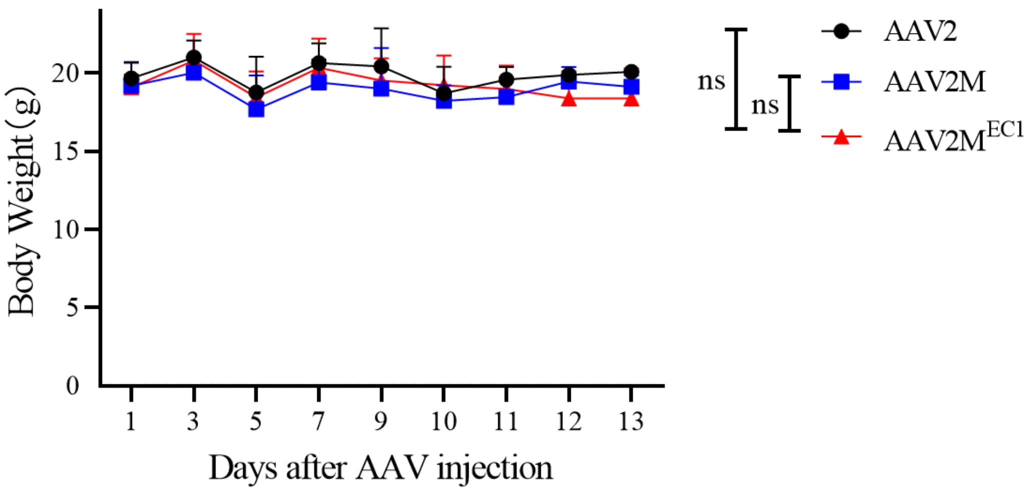

Supplement: Supplementary file 2 — Supplementary Material 2 [file 12885_2023_11705_MOESM2_ESM.pdf]
